# Lipoteichoic Acid Rescued Age‐Related Bone Loss by Enhancing Neuroendocrine and Growth Hormone Secretion Through TLR2/COX2/PGE2 Signalling Pathway

**DOI:** 10.1111/jcmm.70247

**Published:** 2024-12-02

**Authors:** Zixian Liu, Zexin Lin, Yingqi Chen, Mincheng Lu, Weisheng Hong, Bin Yu, Guanqiao Liu

**Affiliations:** ^1^ Department of Orthopaedics, Nanfang Hospital Southern Medical University Guangzhou China; ^2^ Guangdong Provincial Key Laboratory of Bone and Cartilage Regenerative Medicine, Nanfang Hospital Southern Medical University Guangzhou China; ^3^ The Second Hospital and Clinical Medical School Lanzhou University Lanzhou China; ^4^ Department of Orthopedic, Shenzhen Second People's Hospital The First Affiliated Hospital of Shenzhen University Shenzhen China

**Keywords:** bone loss, brain–bone, growth hormone, lipoteichoic acid, PGE2

## Abstract

The phenomenon of brain–bone crosstalk pertains to the intricate interaction and communication pathways between the central nervous system and the skeletal system. Disruption in brain–bone crosstalk, particularly in disorders such as osteoporosis, can result in skeletal irregularities. Consequently, investigating and comprehending this communication network holds paramount importance in the realm of bone disease prevention and management. In this study, we found that *Staphylococcus aureus* lipoteichoic acid promoted the conversion of arachidonic acid to PGE2 by interacting with TLR2 receptors acting on the surface of microglial cells in the pituitary gland, leading to the upregulation of COX‐2 expression. Subsequently, PGE2 bound to the EP4 receptor of growth hormone‐secreting cells and activated the intracellular CREB signalling pathway, promoting GH secretion and ameliorating age‐related bone loss.

## 1 Introduction

Osteoporosis is a severe quality‐of‐life and health hazard characterised by a gradual loss of mineral and integrity of the skeleton, which makes it fragile and susceptible to fracture [1]. Especially in middle‐aged and older adults, bone loss is often accompanied by an increased risk of fracture, which poses a significant health and socioeconomic burden [2, 3]. Current research on osteoporosis focuses on the cells inside the bone marrow cavity [4], which has led to the development of drugs such as bisphosphonates, denosumab and parathyroid hormone to treat osteoporosis [5]. They can improve bone mass by inhibiting osteoclastogenesis and enhancing osteoblast differentiation [6]. However, due to the complex process of osteoporosis pathogenesis, their clinical efficacy is more limited and many factors, such as bone remodelling imbalances, hormone levels, nutritional status, and genetic factors are all thought to be important factors affecting bone density [7, 8]. Therefore, studying osteoporosis from multiple perspectives and emphasising the interregulatory effects among multiple organs will help us better prevent and treat osteoporosis and improve patients' bone health.

Brain–bone axis plays a critical role in bone growth, metabolism and regeneration [9, 10]. Recent studies have shown that neuropeptides and hormones released by the brain regulate the activity of osteoclasts and osteoblasts, which, in turn, affects the process of bone reconstruction [11]. Similarly, the skeleton can act as an endocrine organ, producing biologically active substances such as osteocalcin and leptin, which feed back to the brain and influence appetite and energy metabolism [12, 13]. Overall, various hormones play an essential role in the interconnection of bone and brain. Growth hormone (GH), a protein hormone secreted by the anterior pituitary gland [14], is essential for bone growth and metabolic regulation, and its secretion decreases with age, which leads to a decrease in bone density and bone fragility [15]. Several studies have shown that growth hormone treatment of older adults with osteoporosis enhanced bone density [16, 17]. However, it has also been noted that the direct use of GH for osteoporosis has many side effects, such as arthralgia, muscle pain, oedema and so on [18]. In addition, some studies have shown that the direct use of growth hormone to treat osteoporosis may affect the metabolic regulation of other hormones in the body, for example, insulin resistance [19, 20]. In conclusion, the direct use of growth hormone in treating age‐related bone loss is risky, with higher clinical costs, creating a financial burden on patients [21]. Therefore, searching for factors and pathways indirectly regulating GH secretion is vital to alleviate age‐related bone loss.

Lipoteichoic acid (LTA), one of the critical components of the cell wall of *Staphylococcus aureus* [22], possesses a range of biological activities, including modulation of the immune response [23], influence on host metabolism and so on [24]. These functions are usually considered harmful, and it has been demonstrated that they induce sepsis as well as the development of multiorgan dysfunction in many diseases [25, 26]. However, recent studies have shown that LTA also has a positive effect. The results show that LTA enhanced the body's immune response against tumours or infection, and reduced fat deposition and inflammation in metabolic syndrome and diabetes‐related diseases [27, 28]. Regarding bone metabolism, the study by Kwon et al. [23] states that LTA inhibits osteoclast differentiation and bone resorption via interruption of gelsolin–actin dissociation, thereby ameliorating inflammatory bone loss. Similarly, our study uncovered that LTA could rescue aged‐related bone loss through alleviating macrophage senescence [29]. However, whether GH participated in LTA‐reduced age‐related bone loss is still unknown. Therefore, it is of great value to clarify whether brain–bone crosstalk plays a crucial role in LTA‐induced bone mass and GH secretion.

Prostaglandin E2 (PGE2) is a multifunctional lipid mediator belonging to the prostaglandin family that is produced from arachidonic acid catalysed by cyclooxygenase enzymes (COX‐1 and COX‐2) [30]. PGE2 plays a role in a variety of physiological and pathological processes, including inflammatory responses, pain modulation, gastric mucoprotection, regulation of blood pressure and promotion of platelet aggregation [31, 32]. E‐type prostanoid receptor 4 (EP4), as one of the four known receptors for PGE2 (EP1, EP2, EP3 and EP4), is a G protein–coupled receptor (GPCR) which associated with a variety of signalling pathways, including signalling through the regulation of cAMP levels [33]. Activation of EP4 receptors plays a crucial role in various physiological responses, such as anti‐inflammatory and pro‐inflammatory processes, pain perception, regulation of renal function and bone remodelling [34, 35]. As one of the four known receptors for PGE2, EP4, a G protein–coupled receptor, regulates bone metabolism by modulating multiple signalling pathways, including those that control cAMP levels. Specifically, EP4 activation in osteoclasts is essential for maintaining subchondral bone homeostasis by facilitating osteoclast migration and differentiation. In conditions like osteoarthritis, EP4 expression in osteoclasts contributes to disease progression through increased subchondral bone angiogenesis and sensory neuron innervation, which are associated with pain and joint degeneration [35, 36, 37]. Furthermore, EP4's role extends to the mechanical loading response of bones, where it promotes osteogenesis via a sensory nerve‐mediated pathway that reduces sympathetic tone, thereby supporting bone formation [38]. In summary, EP4 receptors exhibit a dual role in bone metabolism, supporting both bone formation and contributing to bone pathology. While EP4 activation promotes osteogenesis in response to mechanical loading by modulating sympathetic tone, it also drives disease processes in osteoarthritis by enhancing subchondral bone angiogenesis and sensory innervation, which leads to pain and joint degeneration. This dual function highlights the complexity of EP4 signalling in maintaining bone health and its potential as a therapeutic target for bone‐related conditions.

CREB, as a transcription factor, can be activated by various signalling pathways, especially those that lead to elevated intracellular cAMP levels [39]. Once activated, CREB can promote or inhibit the expression of various target genes, thereby affecting cell growth, differentiation and survival [40, 41]. In recent years, several studies have shown that CREB is a key regulator of bone cell activity and plays a complex role in osteoporosis. By influencing osteoblast function and bone formation positively but potentially enhancing osteoclast‐mediated resorption in certain contexts [42, 43, 44, 45], CREB contributes to both bone maintenance and the imbalance seen in osteoporotic bones. Targeting CREB pathways could, therefore, offer therapeutic benefits for osteoporosis by selectively enhancing bone formation while inhibiting excessive bone resorption.

There is a direct correlation between the interaction of PGE2 with EP4 receptors and its further activation of CREB [46]. In cases where PGE2 acts through EP4 receptors, EP4 receptor activation increases the intracellular concentration of cAMP. This increased cAMP activates protein kinase A (PKA) and phosphorylates CREB. Phosphorylated CREB enters the nucleus, binds to specific promoters and promotes targeted gene transcription [46, 47]. This pathway has an important role in various biological processes and pathological states.

In this study, we explored the role of LTA in age‐associated osteoporosis. We found that LTA could activate the TLR2 receptor and increase prostaglandin E2 (PGE2) secretion in microglia by upregulating the expression of cyclooxygenase‐2 (COX‐2). Elevated PGE2 could phosphorate CREB through EP4 and promote the growth hormone secretion through EP4 from adenopituitary cells, which then ameliorates age‐associated bone loss. With this study, we provide new ideas for combating age‐related osteoporosis and theoretical support for antiageing interventions using microbial products.

## 2 Materials and Methods

### 2.1 Mice

In this study, C57BL/6 mice (specific pathogen‐free, weighing 21–27 g, 10 months old) were purchased from the Experimental Animal Center of Southern Medical University (Guangzhou, China). The animals were housed in the Animal Experimental Center of Southern Hospital under controlled conditions with a temperature of 20°C ± 5°C, relative humidity of 50% ± 3% and a 12‐h light–dark cycle. They were provided with food and water ad libitum and observed daily for general conditions, including mental status, food intake and bowel movements.

### 2.2 Cell Lines and Cell Culture

GH3 cell line: Cultured in Ham's F‐12k medium containing 15% horse serum, 2.5% foetal bovine serum and 1% penicillin/streptomycin. Passaging is required when the cell density reaches 80%–90%. BV‐2 cell line: Cultured in DMEM medium containing 10% foetal bovine serum and 1% penicillin/streptomycin. Passaging is required when the cell density reaches 70%–80%.

### 2.3 Animal Experiments

To evaluate the effects of LTA on the age‐related osteoporosis mouse model, the mice were divided into two groups: the LTA group, which received intraperitoneal injections of 200 μL LTA (250 μg/mL) twice a week for 8 weeks, and the control group, which received an equivalent volume of saline. After 8 weeks, bilateral femurs, serum and pituitary glands were collected for further analysis. To assess the impact of celecoxib under LTA treatment on the age‐related osteoporosis mouse model, the mice were divided into three groups. The first two groups were treated as previously described with LTA injections. The celecoxib group received oral gavage of celecoxib (100 mg/kg) 30 min before LTA injection. The celecoxib alone group was given oral celecoxib (100 mg/kg), and their counterparts in the control group received an equivalent volume of saline. After 8 weeks, bilateral femurs, serum and pituitary glands were collected for further analysis.

### 2.4 Micro‐CT Analysis

After blood collection, the femurs from mice were perfused with PBS and subsequently fixed in a paraformaldehyde (PFA) solution. The femurs were immersed in the PFA solution and set on a shaker at 4°C for 1–2 days. Micro‐CT scanning of the femurs was performed with specific parameters, including a voltage of 55 kV, current of 145 mA and integration time of 400 ms. Data analysis was carried out using SkyScan 1176 software, where CTAn was used for region of interest (ROI) analysis, and Dataviewer was used to obtain coronal section two‐dimensional images. Specific analysis parameters included trabecular bone volume fraction (BV/TV), trabecular thickness (Tb.Th), trabecular separation (Tb.Sp), trabecular number (Tb.N), structure model index (SMI), trabecular pattern factor (Tb.Pf), cortical bone thickness (Ct.Th) and cortical bone area (Ct.Ar).

### 2.5 Haematoxylin and Eosin (H&E) Staining and Immunofluorescence

In the context of HE staining, after fixation, the samples were placed in a decalcification solution and decalcified at 4°C for 14 days. Two weeks later, the tissues were dehydrated, embedded in paraffin and sectioned using a microtome with a thickness of 5 μm. HE staining was performed following standard procedures [48]. Bright‐field images were observed under an upright microscope, with an effort to centre the growth plate in the field of view. Images were captured at 4× and 10× magnifications, and the trabecular number was quantified.

For immunofluorescence staining, the samples were processed similar to HE staining up to the decalcification step. After decalcification, the tissues were placed at 4°C, dehydrated in a 20% sucrose solution for 2 days and then blotted dry on filter paper to remove excess sucrose. The bone samples were embedded in OCT at the bottom of a cryomold, with OCT added until filled. The samples were then placed in liquid nitrogen, retrieved after OCT solidified and sectioned along the coronal plane using a cryostat with a thickness of 11–12 μm. The sections were incubated at 37°C for 1 h, washed three times alternately in distilled water and PBST, air‐dried and circled to mark the staining area using an immunohistochemistry pen. Next, the sections were blocked with 5% goat serum at room temperature for 1 h. Subsequently, primary antibodies, GH (1:50), COX‐2 (1:100), Iba‐1 (1:1000) and p‐CREB (1:50), were applied in 40 μL of the prepared primary antibody solution for each sample and incubated overnight at 4°C in a refrigerator. On the following day, the sections were washed three times with PBST and then incubated with a fluorescent secondary antibody (DyLight 594 AffiniPure Goat Anti‐Mouse IgG (1:100), and DyLight 488 AffiniPure Goat Anti‐Rabbit IgG (1:100)) in 5% goat serum at room temperature in the dark for 1 h. After PBST washes, the sections were mounted with neutral resin containing DAPI. Fluorescent images were captured by the BX63 microscope in the dark, with objectives set at 10× and 20× magnifications. Six to eight random fields were selected, and the fluorescence intensity was quantified using Image J software.

### 2.6 ELISA

After isoflurane anaesthesia of mice for approximately 2 min, heart blood was collected and allowed to stand at room temperature for 1–2 h. Afterwards, the samples were centrifuged at 3000 g for 10 min at room temperature to obtain serum. Subsequently, the pituitary gland was isolated, ground in PBS and centrifuged to obtain tissue supernatant. The procedures were carried out following the standardised steps of the assay kit (GH: M150887, Mreda, Beijing, China. PGE2: EK8103/2, Multisciences, Hangzhou, China). Finally, readings were taken using a microplate reader at a wavelength of 450 and 570 nm. The average OD values of all wells were calculated, and the OD value of the zero concentration standard was subtracted. The concentration of the standard samples served as the x‐axis, their corresponding OD values as the y‐axis and regression fitting was performed using Excel software to generate a standard curve. This standard curve was used to determine the concentration of the samples by inputting their OD values, and the final concentration should be multiplied by the dilution factor.

### 2.7 Collection of Conditioned Medium From BV‐2 Cells

The BV‐2 cells were seeded at a density of 10^6^ cells per well in six‐well plates. The culture medium was collected after being stimulated with LTA (1 μg/mL) for 4, 12 and 24 h. After centrifugation at 4000 g for 5 min, the supernatant was filtered through a 0.22‐μm filter and stored at −80°C for future use. In addition, another conditioned medium was collected by seeding BV‐2 cells in six‐well plates and pretreating them with different concentrations of celecoxib (0.5, 2, 10 and 50 μM) for 30 min before adding LTA for 12 h. The subsequent steps were the same as described earlier.

### 2.8 RNA Extraction, Reverse Transcription, Real‐Time Fluorescent Quantitative PCR

BV‐2 and GH3 cells were separately seeded at a density of 10^6^ cells into six‐well plates. For the coculture experiment, BV‐2 and GH3 cells were coinoculated into six‐well plates at a density of 3 × 10^5^ cells each. After the corresponding treatments, cells were collected into centrifuge tubes and centrifuged at 8000 g for 2 min at room temperature, and the supernatant was discarded. After isolation to obtain mouse pituitary tissue, it was stored in liquid nitrogen. RNA extraction was carried out following the standard procedures of the AG company RNA extraction kit (AG21024, Accurate Biology, Changsha, China). Subsequently, RNA concentration was measured using a UV spectrophotometer, and all sample concentrations were diluted to 200 ng/μL with RNase‐free water. Subsequently, reverse transcription was performed on a PCR instrument following the standard procedure of the YEASEN kit (HB210716, YEASEN, Shanghai, China). Briefly, after adding the appropriate reagents, residual genomic DNA was removed by incubating at 42°C for 2 min, followed by reverse transcription. Finally, the reaction system was constructed according to the standard process of the YEASEN company kit (HB210321, YEASEN, Shanghai, China), and amplification detection was performed on the QuantStudio 5 quantitative PCR instrument.

For GH3 cell treatments: GH3 cells were stimulated with different concentrations of LTA (0.1, 1, 10 μg/mL) for 4, 12 and 24 h; conditioned media from BV‐2 cells treated with LTA alone were used to stimulate GH3 cells for 4, 12 and 24 h; PGE2 was used to stimulate GH3 cells at different concentrations (10 nM, 100 nM and 1 μM) for 4, 12 and 24 h; BV‐2 cells were treated with LTA in combination with celecoxib (30 min) before stimulating conditioned media for 12 h; GH3 cells were pretreated with L798106 (200 nM) and E7046 (1 μM) for 30 min, followed by stimulation with PGE2 (100 nM) for 12 h; KG‐501 (10 μM) was pretreated for 30 min, followed by stimulation with PGE2 (100 nM) for 12 h. For coculture treatments: The coculture system was stimulated with LTA (1 μg/mL) for 4, 12 and 24 h. For BV‐2 cell treatments, BV‐2 cells were stimulated with LTA (1 μg/mL) for 4, 12 and 24 h.

### 2.9 Total Protein Preparation and Western Blotting

BV‐2 and GH3 cells were seeded separately into six‐well plates at a density of 1 × 10^6^ cells per well. After the respective treatments, cells were collected into centrifuge tubes and centrifuged at 2500 g at room temperature for 2 min. The supernatant was discarded, and the cells were washed once with PBS, followed by centrifugation to remove the supernatant. The pituitary tissue was washed with PBS and cut into several sections. Then, preprepared cold lysis buffer was added. After incubating on ice for 30 min, the samples were sonicated to dissolve the residue, followed by centrifugation at 12,000 g at 4°C for 15 min. The resulting supernatant was collected as whole cell lysates. Subsequently, the protein concentration was determined using the BCA method. Based on the protein quantification results, 20 μg of total protein was loaded per well for electrophoresis at 120 V for 60 min. Then, the proteins were transferred onto a PVDF membrane at 400 mA/100 V for 90 min. After blocking with 5% skim milk powder for 1 h, the membrane was quickly washed three times with TBST solution and then incubated with primary antibodies overnight at 4°C. The next day, after three rapid washes with TBST solution, HRP‐conjugated anti‐rabbit secondary antibody (1:10,000) or HRP‐conjugated antimouse secondary antibody (1:10,000) were added and incubated for 1 h with gentle agitation on a shaker. Excess liquid on the surface of the PVDF membrane was removed, and the membrane was placed in ECL luminescent liquid, then developed using a BLT developer. Finally, band intensity was quantitatively analysed using ImageJ software.

For BV‐2 cell treatment, C29 (100 μM) was pretreated for 30 min before stimulation with LTA (1 μg/mL) for 4, 12 and 24 h. For GH3 cell treatment, PGE2 (100 nM) was applied for 15, 30 and 60 min. E7046 (10 μM) was pretreated for 30 min before adding PGE2 for 30 min. CAY10580 (10 μM) was used to stimulate GH3 cells for 15, 30 and 60 min. The following primary antibodies were used: COX‐2 (1:1000), p‐CREB (1:1500), T‐CREB (1:1000), β‐Tubulin (1:1000) and GAPDH (1:1000).

### 2.10 Cellular Immunofluorescence

BV‐2 and GH3 cells were separately seeded at a density of 10^4^ cells in 24‐well plates with coverslips. When they reached 70% confluence, they were rinsed three times with PBS. After appropriate treatments, the cells were fixed with 4% paraformaldehyde for 15 min. Subsequently, the coverslips were rinsed three times with PBS, and then 0.2% Triton X‐100 was added for permeabilisation for 5 min. After three more PBS washes, the cells were blocked with 3% BSA for 1 h. The blocking solution was aspirated, and primary antibodies were diluted in antibody dilution buffer and incubated overnight at 4°C. Following this, the coverslips were washed three times with PBST and incubated with appropriate fluorescent secondary antibodies, diluted in antibody dilution buffer, at room temperature in a humid, dark environment for 1 h. After three washes with PBST, the samples were counterstained with DAPI and coverslipped, image acquisition and immunofluorescence steps. For BV‐2 cell treatments: Stimulation with LTA (1 μg/mL) for 4, 12, and 24 h. For GH3 cell treatments: The conditioned medium from BV‐2 cells treated with LTA in combination with celecoxib (10 μM) for 12 h was used for stimulation for 12 h. Pretreatment with KG‐501 (10 μM) for 30 min followed by PGE2 (100 nM) stimulation for 12 h. The following primary and secondary antibodies were used: COX‐2 (1:200) and GH (1:50).

### 2.11 Statistical analysis

In this study, all data results are presented as the mean ± standard error of the mean (Mean ± SD) and were subjected to statistical analysis using GraphPad Prism 8 software. For comparisons between two groups, an independent samples *t*‐test was employed when the sample size was greater than or equal to three, and the data followed a normal distribution. If the variances were equal, a one‐way ANOVA was initially used for statistical analysis. If the ANOVA results were statistically significant, post hoc Bonferroni tests were performed for pairwise comparisons. A *p*‐value less than 0.05 was considered statistically significant, and statistical graphs were generated using GraphPad Prism 8 software. All results in this experiment were obtained from at least three independent control replicate experiments.

### 2.12 List of Experimental Materials and Reagents

Detailed information regarding primer sequences, antibodies, reagents, cell lines and instruments can be found in Figure S2.

## 3 Results

### 3.1 LTA Improves Age‐Related Bone Loss by Increasing the Secretion of Growth Hormone

**FIGURE 1:**
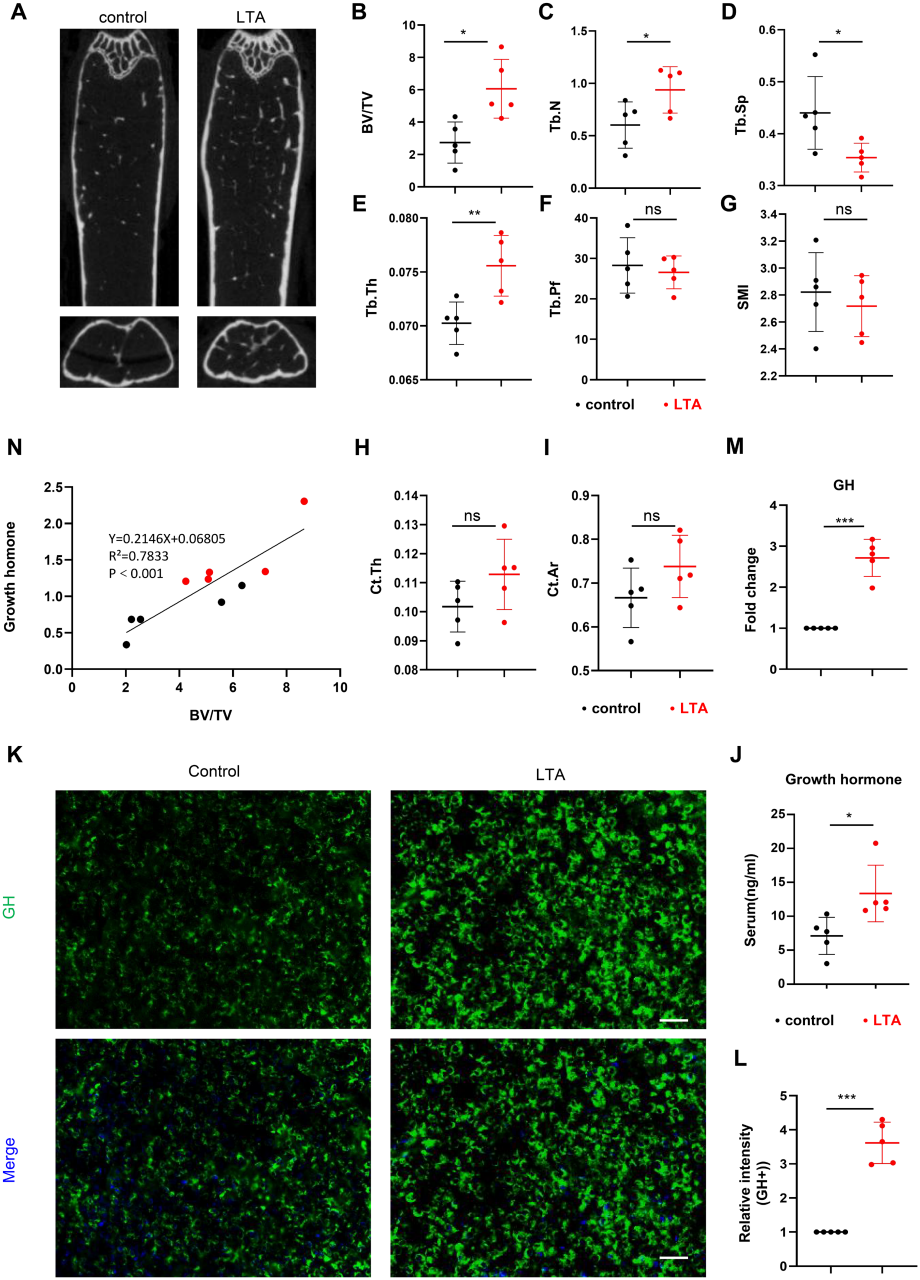
Lipoteichoic acid can increase bone mass in aged mice by regulating growth hormone content. The LTA group received two intraperitoneal injections of 200 μL LTA (250 μg/mL) per week for 8 weeks, while the control group received an equivalent volume of saline. Micro‐CT coronal section representative images of mouse femurs (A), trabecular bone parameter analyses (B–G, *n* = 5): BV/TV (bone volume fraction), Tb.N (trabecular number), Tb.Sp (trabecular separation), Tb.Pf (trabecular pattern factor), Tb.Th (trabecular thickness), SMI (structure model index). Cortical bone parameter analyses (H, I, *n* = 5): Ct.Th (cortical thickness), Ct.Ar (cortical area). ELISA measurement of growth hormone levels in mouse serum (J, *n* = 5). Representative images and quantification of immunofluorescence for GH^+^ cells in the pituitary (green) (K, L, *n* = 5, scale bar = 100 μm). qPCR was used to measure the mRNA expression levels of the GH gene in the pituitary gland (M, *n* = 5). Correlation analysis between serum growth hormone levels and BV/TV (N, *n* = 5). *p < 0.05, **p < 0.01, ***p < 0.001, ns: No statistical difference. Data are presented as mean ± SD. Two‐tailed Student's *t*‐test was used.

To elucidate the role of LTA in age‐related bone loss, we treat 12‐month‐old mice with LTA by intraperitoneal injection. Micro‐CT results demonstrated that in the LTA‐treated group (2 mg/kg) of mice, the bone volume fraction (BV/TV) of the distal trabecular bone of the femur, trabecular number (Tb.N) was significantly higher compared to the control group. In contrast, trabecular separation was reduced considerably (Figure 1A–I).

GH, a protein hormone secreted by the pituitary gland [14], plays a crucial role in the pathogenesis of age‐related osteoporosis [49, 50], contributes to maintaining bone density and quality, promoting bone repair and remodelling, and reducing the risk of osteoporosis [20, 51]. Our ELISA results showed a significant increase in serum GH levels in the LTA‐treated group of mice compared to the control group (Figure 1J), and immunofluorescence staining of the pituitary gland confirmed this finding, with a higher fluorescence intensity of GH in the LTA‐treated group compared with the control group (Figure 1K,L). These results were further confirmed by qPCR, compared to the control group, the GH mRNA level in pituitary tissue significantly increased in the LTA‐treated group (Figure 1M). Furthermore, we observed a strong correlation between the elevated serum growth hormone levels and the increased BV/TV (Figure 1N). These results indicate that LTA rescued age‐related bone loss by enhancing growth hormone secretion in the pituitary gland.

### 3.2 LTA Promotes the Secretion of Growth Hormone by Enhancing COX‐2/PGE2 in the Pituitary Gland

To further explore the mechanism of how LTA increases growth hormone levels, we stimulated GH3 cell lines with different concentrations of LTA. However, compared with the control group, GH gene expression was significantly downregulated in the LTA‐treated group at other time points (Figure S1A). Consistently, ELISA results demonstrated that the GH content in the cell supernatant of the LTA‐treated group was significantly lower than that in the control group (Figure S1B). These results imply that the role of LTA in enhancing GH secretion may be an indirect effect.

**FIGURE 2:**
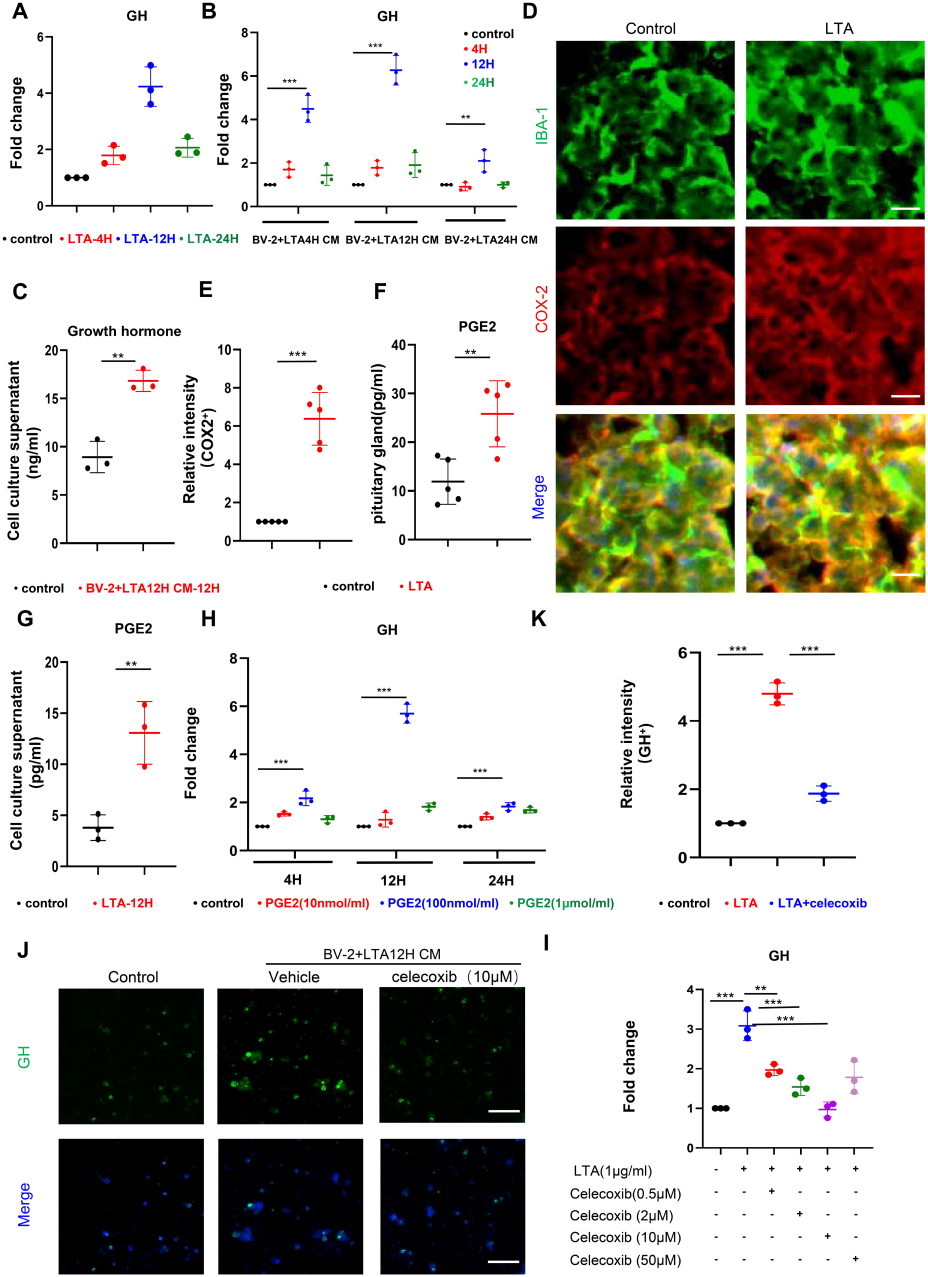
Lipoteichoic acid indirectly promotes the secretion of growth hormone in mice. qPCR was performed to detect the mRNA expression levels of the GH gene in cocultured BV‐2 and GH3 cells after treatment with LTA (1 μg/mL) (A, *n* = 3). qPCR was performed to detect the mRNA expression levels of the GH gene in GH3 cells after stimulation with conditioned medium from BV‐2 cells treated with different concentrations of LTA at different time points (B, *n* = 3). Growth hormone levels were measured in the cell culture supernatant of GH3 cells after stimulation with the supernatant from BV‐2 cells treated with LTA for 12 h (C, *n* = 3). Immunofluorescence images and quantification of Iba1^+^ (green) and COX‐2^+^ (red) cells in the pituitary of the control and LTA groups (D, E, *n* = 5, scale bar = 25 μm). ELISA was used to measure the PGE2 levels in the pituitary tissues of the control and LTA groups (F, *n* = 5). ELISA was used to measure the PGE2 levels in the conditioned medium from BV‐2 cells after 12 h of stimulation with LTA (1 μg/mL) in the control and LTA groups (G, *n* = 3). qPCR was performed to detect the mRNA expression levels of the GH gene in GH3 cells at different time points after stimulation with different concentrations of PGE2 (H, *n* = 3). qPCR was performed to detect the mRNA expression levels of the GH gene in GH3 cells after 12 h of stimulation with conditioned medium from BV‐2 cells, which were pretreated with different concentrations of celecoxib and then stimulated with LTA (I, *n* = 3). Immunofluorescence images and quantification of GH^+^ (green) cells in BV‐2 cell conditioned medium after 12 h of stimulation with LTA, with pretreatment of 10 μM celecoxib (J, K, *n* = 3, scale bar = 50 μm). *p < 0.05, **p < 0.01, ***p < 0.001. Data are presented as mean ± SD. One‐way ANOVA with Tukey's test and two‐tailed Student's *t*‐test were used.

Lipoteichoic acid, as a component of the *Staphylococcus aureus* cell wall [52], is a phosphorylated lipid molecule that can act as an immunogen by binding to specific receptors on immune cells, activating the host's immune response [53, 54]. Recently studies have shown that growth hormone is primarily secreted by the anterior pituitary gland [55], where microglial cells are the predominant immune cell type in this area [55, 56], and their crosstalk may affect the GH secretion. Thus, we cocultured BV‐2 and GH3 cells, treated them with LTA (1 μg/mL) and detected GH expression. The results showed that, compared with the control group, GH expression is upregulated in the LTA‐treated group, with the most significant difference observed at 12 h (Figure 2A).

Meanwhile, we also collected the conditioned medium from the LTA‐treated BV‐2 cells and treated GH3 cells with the conditioned medium. The results showed that, compared to the control group, the conditioned medium generated by LTA (1 μg/mL) stimulation significantly upregulated the expression of the GH gene in GH3 cells at different time points, with the most significant difference observed at 12 h (Figure 2B). The measurement of growth hormone content in the cell supernatant through ELISA further confirms the experimental results mentioned above (Figure 2C).

Recent studies have reported that lipoteichoic acid upregulates the expression of COX‐2 in various cells, such as macrophages, heart cells, pulpal cells, epithelial cells and so on [57]. As the cyclooxygenase family, COX‐2 catalyses arachidonic acid into prostaglandin E2 (PGE2) [58, 59, 60], which has been reported as highly involved in bone metabolism, the results of Su et al. revealed that senescent preosteoclasts activate the COX2‐PGE2 axis and stimulate osteoblast progenitor cell differentiation to promote subchondral bone formation [61]; Jiang et al. [35] found that PGE2/EP4 signalling in osteoclasts mediates angiogenesis in subchondral bone and promotes osteoarthritis progression. In this study, we found that the fluorescence intensity of COX‐2 in the LTA‐treated group was significantly higher in pituitary tissues than in the control group (Figure 2D,E). ELISA analysis showed that, compared with the control group, PGE2 concentrations were significantly elevated in the pituitary gland and cell culture supernatant of the LTA‐treated group (Figure 2F,G). To determine whether PGE2 plays a crucial role in growth hormone secretion, we stimulated GH3 cells with different concentrations of PGE2. The results showed that, compared to the control group, GH mRNA levels increased at different time points after treating PGE2, especially in the 12‐h treatment group (Figure 2H and Figure S1C).

As a COX‐2 inhibitor, celecoxib inhibits the synthesis of PGE2 during the inflammatory process [62]. To determine whether blocking COX‐2 synthesis could reduce PGE2‐depended GH secretion, we treated BV‐2 cells with LTA or combined with different concentrations of celecoxib for 12 h. Conditioned medium was collected and used to stimulate GH3 cells. The results showed that, compared to the LTA‐treated group, the GH mRNA level significantly decreased in the LTA + celecoxib‐treated group (Figure 2I and Figure S1D). Consistent with the mRNA results, the immunofluorescence intensity of GH3 cells in the LTA + celecoxib‐treated group was significantly lower than in the LTA‐treated group (Figure 2J,K). These results indicated that LTA promotes growth hormone secretion by enhancing COX‐2/PGE2 in the pituitary gland.

### 3.3 LTA Promotes COX‐2 Expression by Activating TLR2 Receptors on Microglial Cells

**FIGURE 3:**
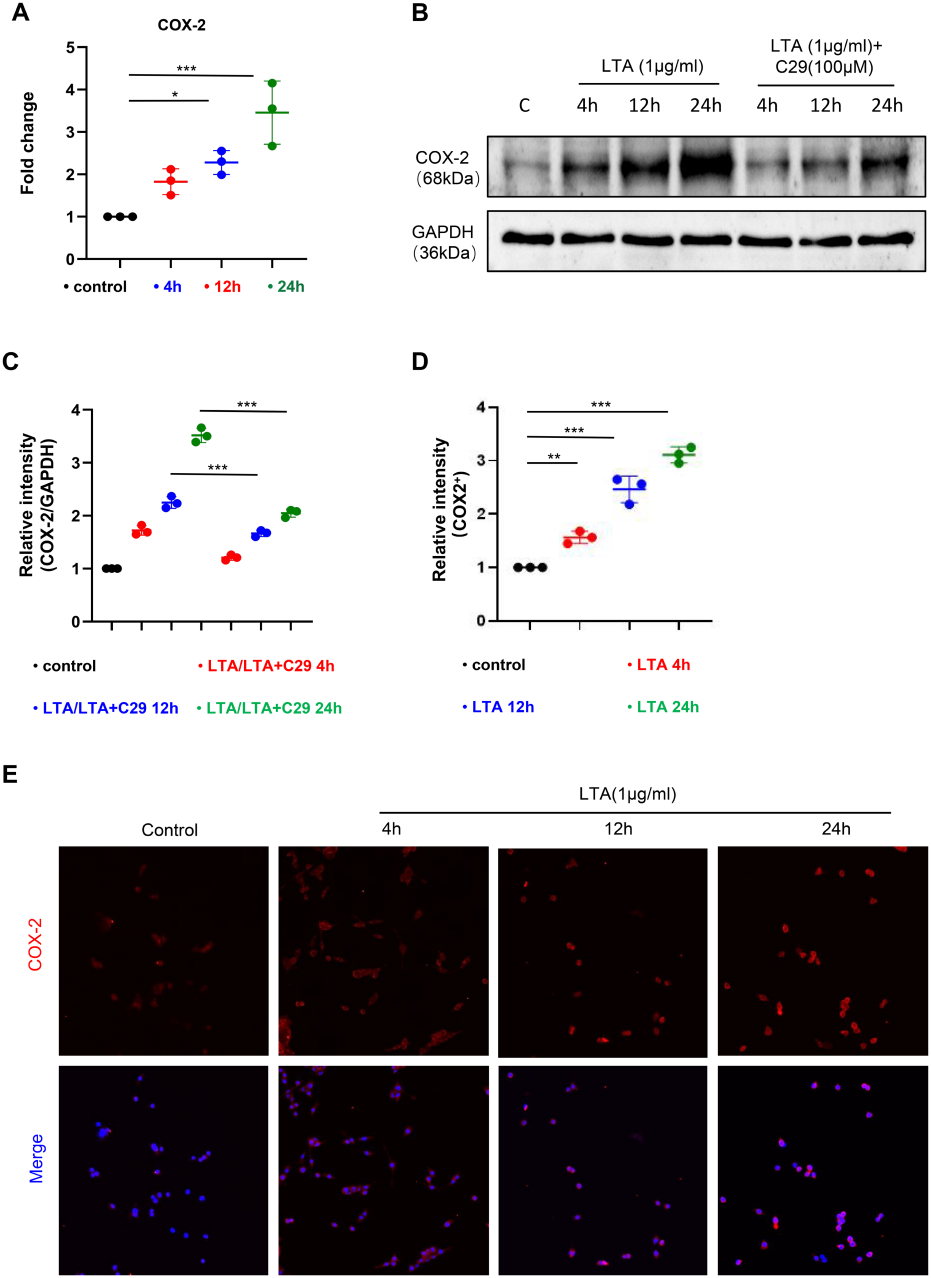
Lipoteichoic acid promotes COX2 expression in microglia via TLR2 thereby promoting PGE2 expression. qPCR was used to measure the mRNA expression levels of the COX‐2 gene at different time points in BV‐2 cells stimulated with LTA (1 μg/mL) (A, *n* = 3). Representative images and quantification of COX‐2 protein expression levels after 15, 30 and 60 min of stimulation with LTA or pretreatment with LTA followed by C29 (100 μM) for 30 min (B, C, *n* = 3). Immunofluorescence images and quantification of COX‐2^+^ (red) cells at different time points in BV‐2 cells stimulated with LTA (D, E, *n* = 3, scale bar = 50 μm). *p < 0.05, **p < 0.01, ***p < 0.001. Data are presented as mean ± SD. One‐way ANOVA with Tukey's test was used.

Previous studies suggest that LTA primarily triggers the immune response in immune cells by binding to Toll‐like receptor 2 (TLR2) receptors [22]. To further elucidate if LTA activates COX‐2 expression through TLR2 in microglial cells, we stimulated BV‐2 cells with LTA or combined with C29 (a TLR2 receptor inhibitor). The results showed that, after LTA treatment, the mRNA level (Figure 3A) and protein level (Figure 3B) of COX‐2 was increased in a time‐dependent manner. Intriguingly, C29 significantly blocked the upregulation of COX‐2 protein levels caused by LTA treatment at the corresponding time points (Figure 3B,C). Similarly, our immunofluorescence staining in BV‐2 cells showed that the fluorescence intensity of COX‐2 in the LTA‐treated group was significantly higher than that in the control group and exhibited a time‐dependent pattern (Figure 3D,E). These results indicated that LTA promotes COX‐2 expression by activating TLR2 receptors on microglial cells.

### 3.4 PGE2 Promotes Growth Hormone Secretion by Activating the EP4/CREB Signalling Pathway

**FIGURE 4:**
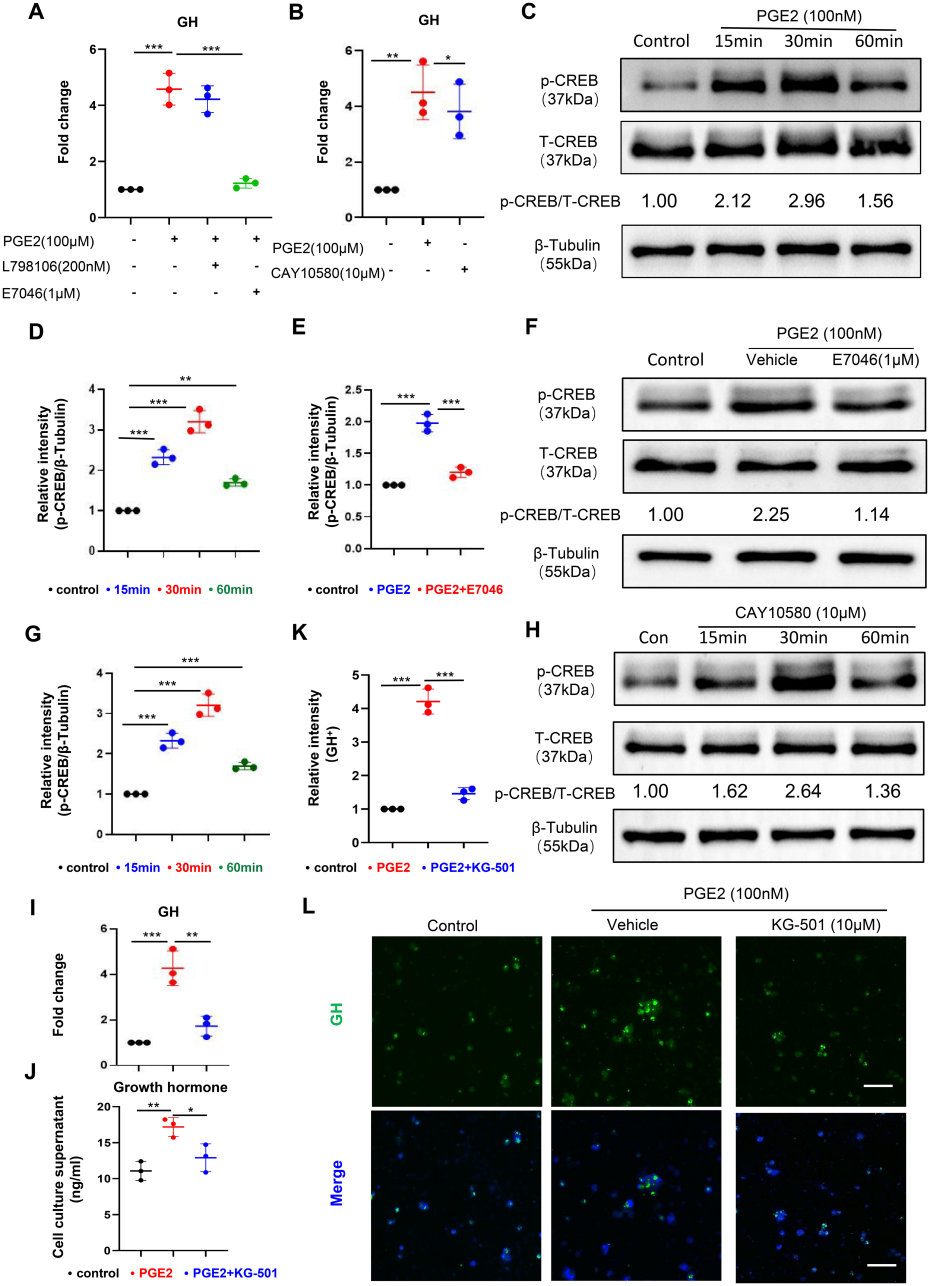
PGE2 promotes the phosphorylation of CREB through the EP4 receptor thereby facilitating GH secretion. qPCR was used to measure the mRNA expression levels of the GH gene in GH3 cells stimulated with PGE2 (100 μM) or PGE2 combined with L98106/E7046 for 12 h (A, *n* = 3). qPCR was used to measure the mRNA expression levels of the GH gene in GH3 cells stimulated with CAY10580 (10 μM) for 12 h (B, *n* = 3). Representative images and quantification of p‐CREB/T‐CREB protein expression levels in GH3 cells stimulated with PGE2 (100 nM) for 15, 30 and 60 min (C, D, *n* = 3). Representative images and quantification of p‐CREB/T‐CREB protein expression levels in GH3 cells stimulated with PGE2 or PGE2 combined with E7046 (1 μM) for 30 min (E, F, *n* = 3). Representative images and quantification of p‐CREB/T‐CREB protein expression levels in GH3 cells stimulated with CAY10580 (10 μM) for 15, 30 and 60 min (G, H, *n* = 3). qPCR was used to measure the mRNA expression levels of the GH gene in GH3 cells stimulated with PGE2 or PGE2 combined with KG501 (10 μM) for 12 h (I, *n* = 3). The concentration of growth hormone in the cell supernatant after stimulation with PGE2 or PGE2 combined with KG501 for 12 h (J, *n* = 3) and representative images and quantification of GH^+^ (green) cells (K, L, *n* = 3, scale bar = 50 μm). *p < 0.05, **p < 0.01, ***p < 0.001. Data are presented as mean ± SD. One‐way ANOVA with Tukey's test was used.

PGE2 regulates various biological effects by targeting its specific receptor. Currently, four major types of EP receptors have been identified: EP1, EP2, EP3 and EP4. EP3 and EP4 receptors are the two most extensively studied subtypes [63] and play crucial roles in various physiological and pathological conditions such as immunity [64], inflammation [65], nervous system function [66, 67] and pain [31, 68]. To determine how PGE2 promotes growth hormone secretion, we separately treated GH3 cells with PGE2 or combined with L984106 (EP3 receptor inhibitor) and E7046 (EP4 receptor inhibitor). The results showed that the EP4 inhibitor significantly blocked the effect of PGE2 on elevating GH levels, whereas the EP3 inhibitor had no blocking effect (Figure 4A). To further determine the role of EP4 in PGE‐2‐induced GH secretion, we used CAY10580 (EP4 receptor agonist) instead of PGE2 to stimulate GH3 cells. The results showed that the CAY10580 treatment significantly upregulated GH gene expression compared to the control group (Figure 4B), which has the same effect as PGE2. These results indicated that PGE2 increases the GH secretion through EP4 receptors.

Previous studies suggested that the cAMP‐PKA‐CREB signalling pathway can influence GH secretion [69, 70]. To elucidate whether PGE2/EP4 regulated GH secretion through the cAMP‐PKA‐CREB signalling pathway. We detected p‐CREB and *t*‐CREB levels after treating the GH3 cells with PGE2. Western blot results showed that, compared to the control group, the PGE2‐treated group exhibited a significant increase in p‐CREB expression levels, peaking at 30 min (Figure 4C,D).

To clarify the role of the EP4 receptor in this process, we treated GH3 cells with PGE2 or combined with EP4 inhibitor. Western blot results showed that, compared to the PGE2‐treated group, elevated p‐CREB expression level caused by PGE2 was significantly blocked by EP4 inhibitor (Figure 4E,F). Additionally, when we used CAY10580 instead of PGE2 to stimulate GH3 cells, western blot results showed that the CAY10580‐treated group exhibited a significant increase in p‐CREB expression levels compared to the control group, peaking at 30 min, consistent with the earlier findings (Figure 4G,H). These results indicated that p‐CREB may be involved in PGE2/EP4‐regulated GH secretion.

Furthermore, we verified whether KG‐501 (CREB inhibitor) could block the PGE2‐induced increase in GH gene expression. qPCR results showed that, compared to the PGE2‐treated group, elevated GH mRNA expression caused by PGE2 stimulation was significantly blocked by CREB inhibitor (Figure 4I). Meanwhile, the GH concentration (Figure 4J) and fluorescence intensity (Figure 4K,L), compared with the PGE2‐treated group, are all significantly lower in the PGE2 + EP4 inhibitor‐treated group. These results indicated that PGE2 enhances CREB phosphorylation through the EP4 receptors, promoting growth hormone secretion.

### 3.5 Celecoxib Blocked the Increased Growth Hormone Secretion and Bone Mass Induced by LTA

**FIGURE 5:**
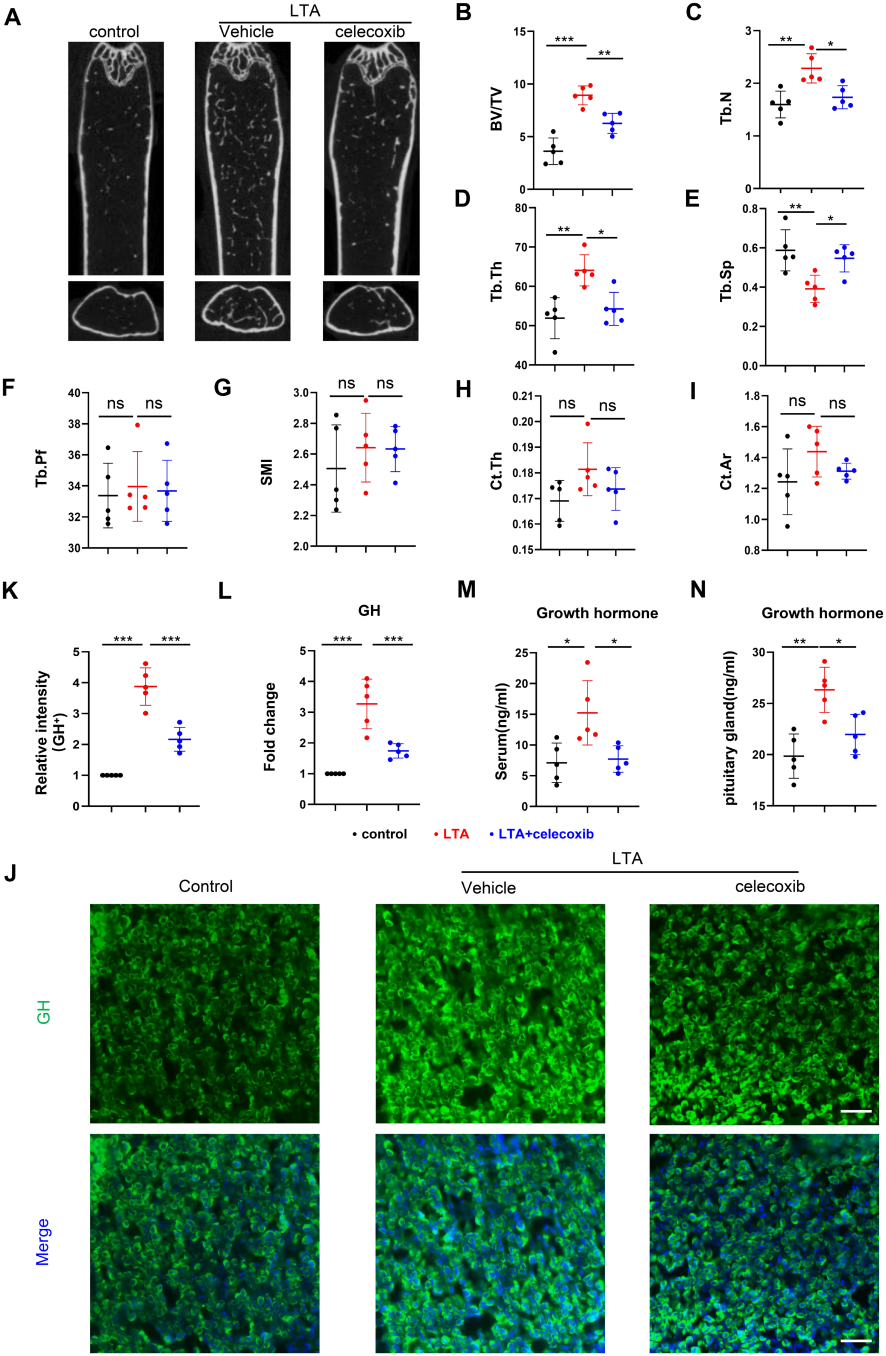
Celecoxib blocked the increased growth hormone secretion and bone mass induced by LTA. The LTA group received a 200‐μL intraperitoneal injection of LTA (250 μg/mL) twice a week for 8 weeks. The celecoxib group orally received celecoxib (100 mg/kg) 30 min before LTA injection. The control group received an equivalent volume of physiological saline. Representative Micro‐CT coronal sections of mouse femurs (A), trabecular bone parameter analysis (B–G, *n* = 5): BV/TV (bone volume/tissue volume), Tb.N (trabecular number), Tb.Sp (trabecular separation), Tb.Pf (trabecular pattern factor), Tb.Th (trabecular thickness), SMI (structure model index). Cortical bone parameter analysis (H, I, *n* = 5): Ct.Th (cortical thickness), Ct.Ar (cortical area). Representative images and quantification of GH^+^ (green) cells in the pituitary gland (J, K, *n* = 5, scale bar = 50 μm). qPCR was used to measure the mRNA expression levels of the GH gene in the pituitary gland (L, *n* = 5). ELISA measurements of growth hormone levels in mouse serum and pituitary tissue (M, N, *n* = 5). *p < 0.05, **p < 0.01, ***p < 0.001, ns: No statistical difference. Data are presented as mean ± SD. One‐way ANOVA with Tukey's test was used.

To elucidate if inhibited COX‐2 activity and PGE2 synthesis could block the increased growth hormone and bone formation induced by LTA, we treated C57BL/6J mice with LTA (2 mg/kg) intraperitoneally or combined with celecoxib (100 mg/kg) orally. Micro‐CT results showed that compared to the LTA‐treated group, the LTA + celecoxib‐treated group exhibited significantly lower trabecular bone volume fraction (BV/TV), trabecular number (Tb.N), trabecular thickness (Tb.Th), cortical bone thickness (Ct.Th) and cortical bone area (Ct.Ar) at the distal end of the mouse femur. In contrast, trabecular separation (Tb.Sp) was significantly higher (Figure 5A–I). ELISA results indicated that the levels of GH in the serum and pituitary tissue homogenates of the LTA + celecoxib‐treated group were substantially lower than those in the LTA‐treated group (Figure 5M,N). qPCR results showed that, compared to the LTA‐treated group, the GH mRNA level in pituitary tissue significantly decreased in the LTA + celecoxib‐treated group (Figure 5L). These results were further confirmed by immunofluorescence staining of the pituitary, where the fluorescence intensity of GH in the LTA + celecoxib‐treated group was significantly lower than in the LTA‐treated group (Figure 5J,K). In addition, we verified that celecoxib treatment alone in mice does not increase the amount of growth hormone in pituitary tissue and serum (Figure S1E–H). These results indicated that celecoxib blocked the increased growth hormone secretion and bone mass induced by LTA.

### 3.6 Celecoxib Blocked the p‐CREB Elevation Induced by LTA

**FIGURE 6:**
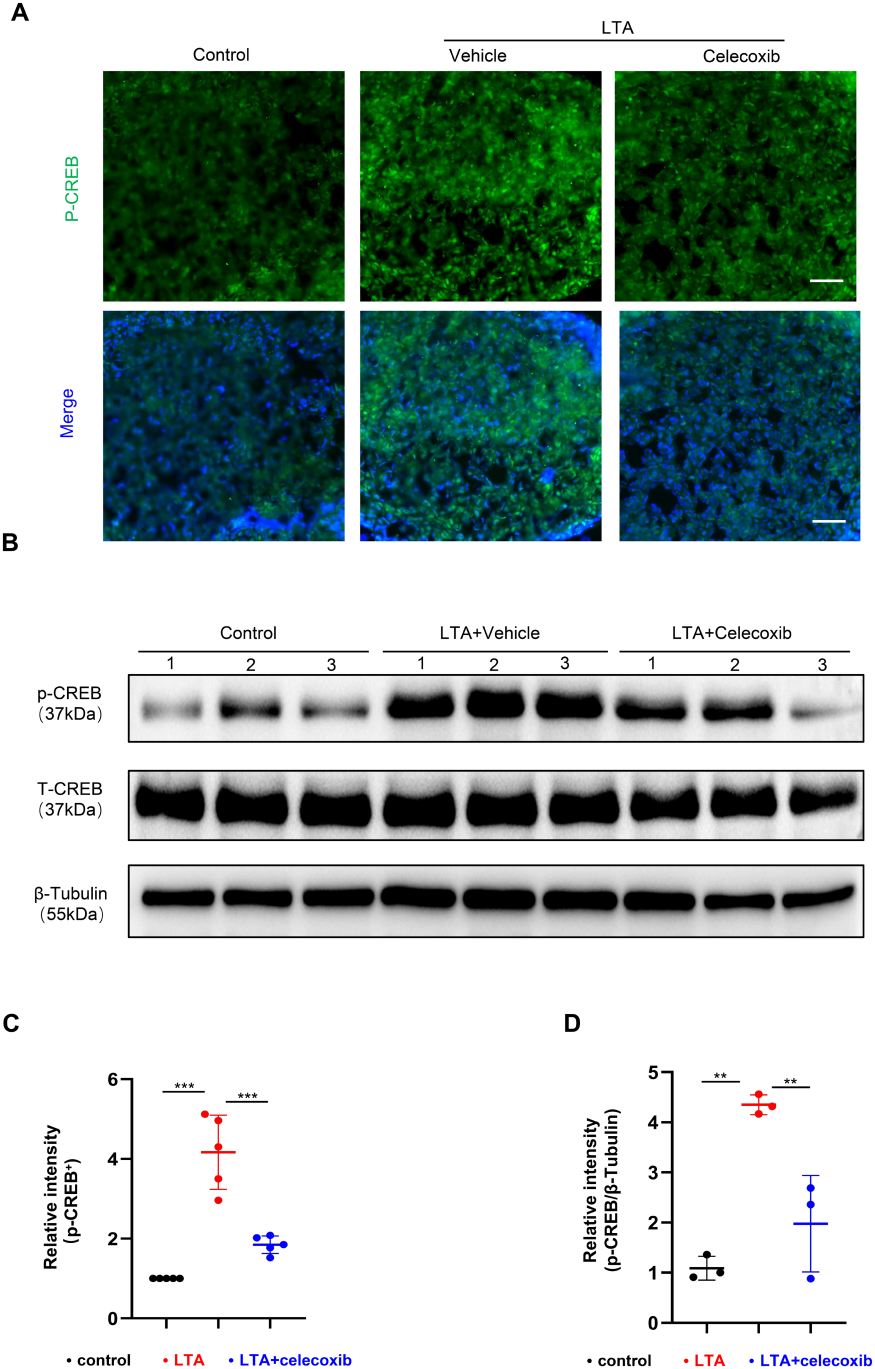
Celecoxib blocked the p‐CREB elevation induced by LTA. Immunofluorescence representative images and quantification of p‐CREB^+^ (green) cells in the pituitary gland of the control group, LTA group and LTA combined with celecoxib group (A, B, *n* = 5, scale bar = 50 μm). Representative images and quantification of p‐CREB/T‐CREB protein expression levels in the pituitary gland of the control group, LTA group and LTA combined with celecoxib group (C, D, *n* = 3). **p < 0.01, ***p < 0.001. Data are presented as mean ± SD. One‐way ANOVA with Tukey's test was used.

To validate the role of the CREB signalling pathway in this process, immunofluorescence staining of the pituitary revealed that the fluorescence intensity of p‐CREB was significantly lower in the LTA + celecoxib‐treated group compared to the LTA‐treated group (Figure 6A,B). Furthermore, we extracted proteins from pituitary tissue. Western blot results showed that, compared to the control group, the LTA‐treated group exhibited a significant increase in p‐CREB expression levels, whereas LTA + celecoxib‐treated group was significantly blocked the p‐CREB elevation induced by LTA (Figure 6C,D). These results indicated that celecoxib partially blocked the LTA‐induced increase in PGE2 levels, reducing p‐CREB expression in pituitary tissue and decreasing GH secretion and bone loss.

## 4 Discussion

**FIGURE 7:**
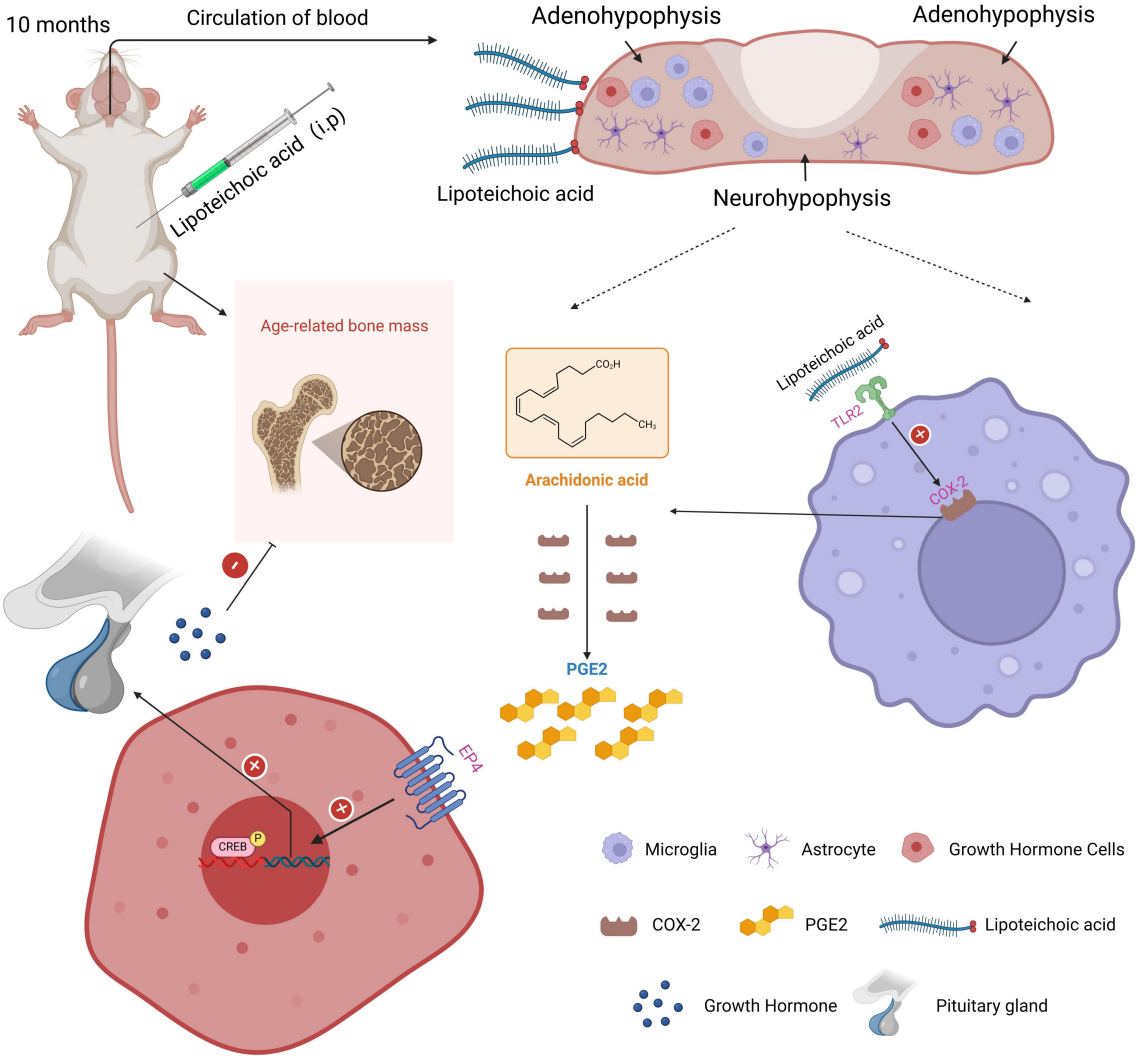
LTA ameliorates age‐related bone loss by activating the TLR2‐COX‐2‐PGE2 axis in pituitary microglial cells, enhancing GH secretion via the EP4‐CREB pathway.

This study identified the critical roles of lipoteichoic acid (LTA) in age‐induced bone loss. Firstly, we found that LTA could elevate the secretion of GH in aged mice and its concentration was correlated with the increase in bone mass. However, in vitro study, we found treating GH3 cells with LTA decreased GH secretion, which indicates that the role of LTA in enhancing GH secretion may not be targeted on GH3 cells directly. As a component of the *Staphylococcus aureus* cell wall [22, 52], LTA acts as an immunogen and binds to specific receptors on immune cells, which activates the host's immune response [53, 54]. Thus, we used LTA to stimulate BV‐2 cells (microglia cell line) and collected the conditioned medium. We found that GH secretion increased after treating GH3 cells with the conditioned medium, indicating that microglia‐GH crosstalk may be crucial in LTA‐induced GH secretion. Furthermore, we enclosed the mechanism of LTA‐induced GH secretion. LTA enhanced COX‐2 activity and PGE2 synthesis in BV‐2 cells, and PGE2 secreted by BV‐2 cells could induce GH secretion in GH3 cells through the cAMP‐PKA‐CREB signalling pathway. Therefore, it is concluded that LTA caused the elevated secretion of GH via microglia‐derived PGE2 to protect against bone destruction in age‐related osteoporosis (Figure 7).

Osteoporosis is one of the most common systemic metabolic diseases associated with inflammation and ageing worldwide [2, 3]. Although many studies have revealed that osteoporosis is characterised by the imbalance between bone resorption by osteoclasts and bone formation by mesenchymal lineage osteoblasts within the bone [1], the cross‐linking relationship in the pathogenesis process of osteoporosis is unknown. Currently, there are many side effects along with the limited efficacy of the drugs used to treat osteoporosis in the clinic. Therefore, studying the pathogenesis of osteoporosis from more perspectives will help us to obtain more therapeutic means in the clinic. With the profound exploration of the nervous neuroendocrine system, it has been further revealed that the ‘brain‐bone’ axis may be a potential target for the occurrence and progression of osteoporosis [9]. Therefore, learning and understanding the relationship between brain and bone may become another direction for treating osteoporosis.

The level of growth hormone production in the body decreases with age and is relatively low in people over the age of 60 [15]. Recent studies have shown that GH deficiency is associated with low bone mass [71]. Our study confirmed that bone mass positively correlated with growth hormone secretion in aged mice, which suggested that GH is a potential benefit factor in osteoporosis.

Microglia, one of the most common innate immune cells in the central nervous system (CNS), participate in CNS development, homeostasis and nearly all CNS disturbances [72]. Recent studies have shown that the inducible COX‐2/PGE2 axis in microglia cells is a pivotal feature in modulating functions of CNS [73]. Our findings showed that microglia‐derived PGE2 was increased in response to LTA stimulation, which induced growth hormone cells to secret GH in CNS. These findings indicate that elevated microglia‐derived PGE2 is the main reason for the secretion of GH rather than LTA direct stimulation. It is shown that PGE2, as one of the inflammatory factors secreted by microglia cells, led to phosphorylation of CREB by EP4 [73, 74]. Consistently, we found that PGE2 induced GH elevation via promoting the expression of EP4 and phosphorylation of CREB in growth hormone cells, which is blocked by E7064 (EP4 inhibitors), indicating the role of the PGE2‐EP4‐CREB axis in the secretion of GH. This pathway highlights how brain‐derived signals can regulate bone processes, specifically through the modulation of GH secretion. While our findings focus on this brain‐to‐bone signalling pathway, other studies have identified the reverse interaction—where bone‐derived signals influence brain health. For example, PDGF‐BB, secreted by preosteoclasts in bone, has been shown to induce cerebrovascular calcification via the p‐ERK/RUNX2 signalling pathway in cerebral vascular cells, resulting in osteogenic changes and mineral deposition in the CNS vasculature [75]. Similarly, osteocalcin, a hormone produced by osteoblasts, has been shown to cross the blood–brain barrier and influence cognitive functions by enhancing hippocampal‐dependent memory and reducing anxiety [76, 77]. Furthermore, studies have indicated that sclerostin, a glycoprotein secreted by osteocytes, may also impact brain health by modulating Wnt signalling, which is crucial for both bone formation and neurogenesis. Reduced levels of sclerostin have been associated with enhanced neurogenesis and cognitive function, suggesting that this bone‐derived signal might play a role in CNS homeostasis and age‐related cognitive resilience [78, 79]. These examples underscore a bidirectional relationship between the brain and bone, where brain‐derived signals modulate bone function, while bone‐derived factors impact neurovascular and neurocognitive health. Together, these findings suggest a complex interplay between the CNS and skeletal system, providing valuable insights into potential therapeutic targets for managing neuroendocrine, neurovascular and neurocognitive aspects of ageing.

Lipoteichoic acid is an important cell wall polymer found in gram‐positive bacteria [22]. It has been demonstrated that a well‐known TLR2 ligand LTA can activate microglial cells and produce various pro‐inflammatory cytokines [22, 80]. Our study has shown that LTA activated microglia cells via TLR2, promoting COX2 expression. It is well known that COX2 can catalyse the conversion of arachidonic acid to PGE2 [59, 81]. Thus, in this study, to clarify the relationship between COX‐2 and PGE2, C29 (inhibitors of COX2) was used. As a result, we demonstrated that C29 attenuated microglia‐derived PGE2 produced by LTA stimulation, suggesting that LTA may induce microglia‐derived PGE2 through the TLR2‐COX2 signalling pathway in CNS. Thus, the role of the COX‐2/PGE2 axis in immune responses is central to the current study. Beyond our findings, other studies have demonstrated that the COX‐2/PGE2 axis plays broader roles in bone health, particularly through its involvement in inflammatory processes that impact bone remodelling and repair. For instance, in metabolic syndrome‐associated osteoarthritis (MetS‐OA), this pathway has been shown to promote subchondral bone changes by driving the senescence‐associated secretory phenotype (SASP) in preosteoclasts. The secreted factors from these senescent cells stimulate osteoblast differentiation and enhance subchondral bone formation, which accelerates joint degeneration in osteoarthritis [61]. In the context of bone fractures, another study found that pro‐inflammatory M1 macrophages can enhance osteogenesis through the COX‐2/PGE2 pathway by promoting mineralisation in mesenchymal stem cells (MSCs) [82]. This finding, in contrast to the pathological remodelling in MetS‐OA, indicates a dual role of COX‐2/PGE2 in supporting bone repair and regeneration, potentially highlighting a context‐dependent effect of this axis. Moreover, research on BMP‐2 signalling in chondrocytes shows that BMP‐2 upregulates COX‐2 expression, which subsequently leads to ATF4 phosphorylation and enhances osteogenesis through EP4 receptor activation [83]. This pathway demonstrates a molecular mechanism where COX‐2/PGE2 not only mediates inflammation but also directly promotes osteogenic signalling. Taken together, these studies illustrate that the COX‐2/PGE2 axis plays multifaceted roles across various bone contexts—from metabolic diseases to fracture repair—suggesting its therapeutic potential as a regulator of bone health and remodelling processes.

It has been shown that LTA influences bone metabolism by direct regulation of bone cells and indirect regulation via immunomodulation [23]. However, the molecular details by which LTA affects bone metabolism remain unclear. In this present study, we revealed a novel regulatory role of LTA in age‐related bone loss. This study demonstrated that LTA induced growth hormone cells to secrete GH via activating microglia cells. Meanwhile, similar to other studies, we also demonstrate that elevated GH may protect against bone destruction in aged mice.

## Author Contributions

**Zixian Liu:** data curation (equal), formal analysis (lead), validation (equal), visualization (equal), writing – original draft (lead). **Zexin Lin:** data curation (equal), validation (equal), visualization (equal). **Yingqi Chen:** data curation (supporting). **Mincheng Lu:** data curation (supporting). **Weisheng Hong:** data curation (supporting). **Bin Yu:** conceptualization (equal), funding acquisition (equal), methodology (equal), project administration (equal), supervision (equal), writing – review and editing (equal). **Guanqiao Liu:** conceptualization (equal), funding acquisition (equal), methodology (equal), project administration (equal), supervision (equal), writing – review and editing (equal).

## Conflicts of Interest

The authors declare no conflicts of interest.

## Supporting information


**Figure S1.** qPCR was performed to detect the mRNA expression levels of the GH gene in GH3 cells after direct treatment with different concentrations of LTA (A, *n* = 3). Growth hormone levels were measured in the cell culture supernatant of GH3 cells stimulated with different concentrations of LTA (B, *n* = 3). Growth hormone levels were measured in the cell culture supernatant of GH3 cells stimulated with PGE2 (100 nM) for 12 h (C, *n* = 3). Growth hormone levels were measured in the cell culture supernatant of GH3 cells after stimulation with the supernatant from BV‐2 cells pretreated with 10 μM celecoxib and then exposed to LTA for 12 h (D, *n* = 3). The celecoxib group orally received celecoxib (100 mg/kg). The control group received an equivalent volume of physiological saline. Representative images and quantification of GH^+^ (green) cells in the pituitary gland (E, F, *n* = 5, scale bar = 50 μm). ELISA measurements of growth hormone levels in mouse serum and pituitary tissue (G, H, *n* = 5). *p < 0.05, **p < 0.01, ***p < 0.001. Data are presented as mean ± SD. One‐way ANOVA with Tukey’s test and two‐tailed Student’s *t*‐test were used.
**Figure S2.** The primer sequence, antibody, reagent, cell line and instrument information.

## Data Availability

All data presented in the current study are available from the corresponding author on reasonable request.
