# Supplementary material for: Lipoteichoic Acid Rescued Age‐Related Bone Loss by Enhancing Neuroendocrine and Growth Hormone Secretion Through TLR2/COX2/PGE2 Signalling Pathway
Source: J Cell Mol Med. 2024 Dec 2;28(23):e70247. doi: 10.1111/jcmm.70247 (PMC11611525; doi:10.1111/jcmm.70247)
Supplement: Supplementary file 1 — Figure S1. qPCR was performed to detect the mRNA expression levels of the GH gene in GH3 cells after direct treatment with different concentrations of LTA (A, n = 3). Growth hormone levels were measured in the cell culture supernatant of GH3 cells stimulated with different concentrations of LTA (B, n = 3). Growth hormone levels were measured in the cell culture supernatant of GH3 cells stimulated with PGE2 (100 nM) for 12 h (C, n = 3). Growth hormone levels were measured in the cell culture supernatant of GH3 cells after stimulation with the supernatant from BV‐2 cells pretreated with 10 μM celecoxib and then exposed to LTA for 12 h (D, n = 3). The celecoxib group orally received celecoxib (100 mg/kg). The control group received an equivalent volume of physiological saline. Representative images and quantification of GH+ (green) cells in the pituitary gland (E, F, n = 5, scale bar = 50 μm). ELISA measurements of growth hormone levels in mouse serum and pituitary tissue (G, H, n = 5). *p < 0.05, **p < 0.01, ***p < 0.001. Data are presented as mean ± SD. One‐way ANOVA with Tukey’s test and two‐tailed Student’s t‐test were used. Figure S2. The primer sequence, antibody, reagent, cell line and instrument information. [file JCMM-28-e70247-s001.pdf]

Figure S1

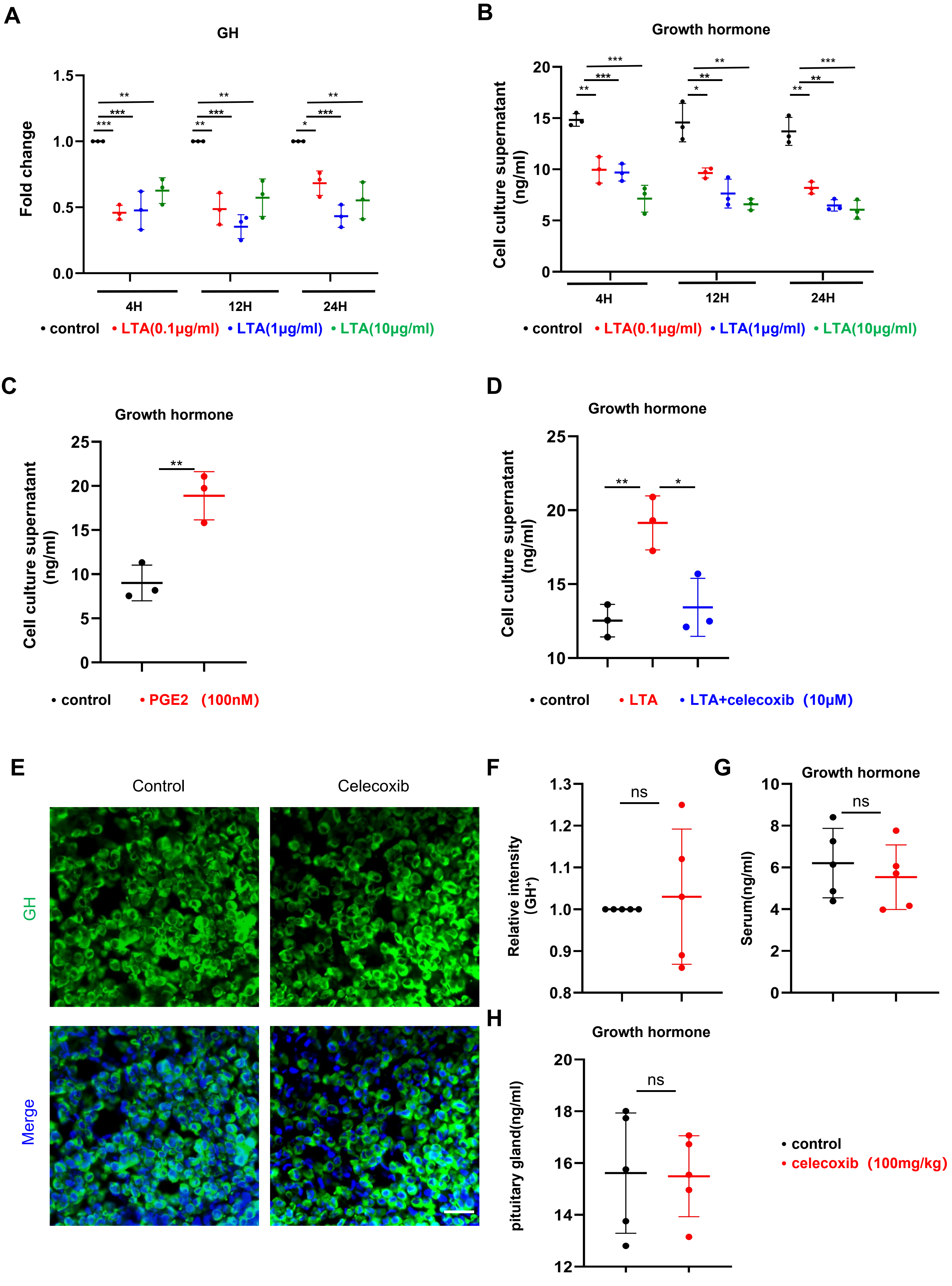
A
GH
Fold change
1.5
1.0
0.5
0.0
***
**
*
4H
12H
24H
• control • LTA(0.1µg/ml) • LTA(1µg/ml) • LTA(10µg/ml)
B
Growth hormone
Cell culture supernatant (ng/ml)
20
15
10
5
0
4H
12H
24H
• control • LTA(0.1µg/ml) • LTA(1µg/ml) • LTA(10µg/ml)
C
Growth hormone
Cell culture supernatant (ng/ml)
25
20
15
10
5
0
**
• control • PGE2 (100nM)
D
Growth hormone
Cell culture supernatant (ng/ml)
25
20
15
10
**
*
• control • LTA • LTA+celecoxib (10µM)
E
Control
Celecoxib
GH
Merge
F
Relative intensity (GH+)
1.3
1.2
1.1
1.0
0.9
0.8
ns
G
Growth hormone
Serum(ng/ml)
10
8
6
4
2
0
ns
H
Growth hormone
pituitary gland(ng/ml)
20
18
16
14
12
ns
• control
• celecoxib (100mg/kg)

Figure S2

The primer sequences are as follows:

Gene	Forward primer（5’-3’）	Reverse primer（3’-5’）
GAPDH	AGGTCGGTGTGAACGGATTTG	TGTAGACCATGTAGTTGAGGTCA
GH	TACAAAGAGTTCGAGCGTGC	CGAGAAGCGAAGCAATTCCA
COX-2	TTCAACACACTCTATCACTGGC	AGAAGCGTTTGCGGTACTCAT

Antibodies information are as follows:

Antibody	Item No. & Company
GH	RPA044Mu01, Cloud-Clone, Wuhan, China
COX-2	66351-1, Proteintech, Guangzhou, China
Iba-1	ab178847, Abcam, USA
p-CREB	28792-1, Proteintech, Guangzhou, China
T-CREB	PTM-5810, PTMBIO, Hangzhou, China
β-Tubulin	EM0103, Huabio, Hangzhou, China
GAPDH	ET1601-4, Huabio, Hangzhou, China
DyLight 594 AffiniPure Goat Anti-Mouse IgG	H+L; A23410; Abbkine, China
DyLight 488 AffiniPure Goat Anti-Rabbit IgG	H+L; A23220; Abbkine, China
HRP-conjugated anti-rabbit secondary antibody	HA1001, Huabio, Hangzhou, China
HRP-conjugated anti-mouse secondary antibody	HA1006, Huabio, Hangzhou, China

Reagents and cell lines information are as follows:

Antibody	Item No. & Company
LTA	L2515, Sigma, USA
Celecoxib	M026597, Mreda, Beijing, China
DAPI	62248; Thermo Scientific™, Shanghai, China
L798106	HY-15274, MCE, USA
E7046	HY-103088, MCE, USA
PGE2	HY-101952, MCE, USA
KG-501	HY-103299, MCE, USA
CAY10580	16835, Cayman, USA
GH3 cell line	CL-0340, Procell, Shenzhen, China
BV-2 cell line	KG645, KEYGEN, Guangzhou, China

Instruments information are as follows:

Instrument	Item No. & Company
Micro-CT	Scanco Medical, Wangen-Bruttisellen, Switzerland
BX63 microscope	Olympus, Tokyo, Japan
CX23 microscope	Olympus, Tokyo, Japan
UV spectrophotometer	AZY2122465, Thermo Fisher, USA
PCR instrument	UNO II Thermoblock, Biometra, Germany
QuantStudio 5 quantitative PCR instrument	Thermo Fisher, USA
BLT	Gelview 6000 Pro, Guangzhou, China
